# Identification of the lncRNA–miRNA‒mRNA regulatory network for middle cerebral artery occlusion-induced ischemic stroke

**DOI:** 10.3389/fgene.2023.1169190

**Published:** 2023-05-09

**Authors:** Guixin Shi, Dong He, Hua Xiao, Yu’e Liu, Chuanyong Liu, Fang Cao

**Affiliations:** ^1^ Department of Neurosurgery, Affiliated Hospital of Zunyi Medical University, Zunyi, China; ^2^ Tongji University Cancer Center, Shanghai Tenth People’s Hospital of Tongji University, School of Medicine, Tongji University, Shanghai, China; ^3^ Dingtao District Hospital of Traditional Chinese Medicine, Heze, China

**Keywords:** MCAO, mRNA, miRNA, lncRNA, ischemic stroke

## Abstract

Stroke known as a neurological disease has significant rates of disability and mortality. Middle cerebral artery occlusion (MCAO) models in rodents is crucial in stroke research to mimic human stroke. Building the mRNA and non-conding RNA network is essential for preventing MCAO-induced ischemic stroke occurrence. Herein, genome-wide mRNA, miRNA, and lncRNA expression profiles among the MCAO group at 3 h, 6 h, and 12 h after surgery and controls using high-throughput RNA sequencing. We detected differentially expressed mRNAs (DE-mRNAs), miRNAs (DE-miRNAs), and lncRNAs (DE-lncRNAs) between the MCAO and control groups. In addition, biological functional analyses were conducted, including GO/KEGG enrichment analysis, and protein-protein interaction analysis (PPI). GO analysis indicated that the DE-mRNAs were mainly enriched in several important biological processes as lipopolysaccharide, inflammatory response, and response to biotic stimulus. The PPI network analysis revealed that the 12 DE-mRNA target proteins showed more than 30° with other proteins, and the top three proteins with the highest node degree were Alb, IL-6, and TNF. In the DE-mRNAs, we found the mRNA of Gp6 and Elane interacting with two miRNAs (novel_miR_879 and novel_miR_528) and two lncRNAs (MSTRG.348134.3 and MSTRG.258402.19). As a result of this study, a new perspective can be gained into the molecular pathophysiology leading to the formation of MCAO. The mRNA-miRNA‒lncRNA regulatory networks play an important role in MCAO-induced ischemic stroke pathogenesis and could be applied to the treatment and prevention of ischemic stroke in the future.

## 1 Introduction

As an acute cerebral vascular disease, stroke is a leading cause of hospitalization for neurologic disease and long-term disability, with the majority (>80%) being ischemic stroke (an abrupt blockage of an artery) (Stegner et al., 2019; Shekhar et al., 2021; Fang et al., 2022). During an ischemic stroke, blood flow to the brain is decreased, triggering cascading events that eventually result in cell death (Mehta et al., 2007; Lai et al., 2011; Chen et al., 2022). Since more than 80% of ischemic strokes occur in the territory of middle cerebral artery (MCA), experimental focal cerebral ischemia models including middle cerebral artery occlusion (MCAO) modelsare crucial in stroke research to mimic human stroke, which causes focal cerebral hypoperfusion and leads to ischemic stroke. The main therapeutic approach for MCAO-induced ischemic stroke is to restore or supply enough fresh blood flow to the brain by reperfusion. However, due to excitotoxicity and oxidative damage, side effects such as ischemic/reperfusion (I/R) may happen after cerebral ischemia and cerebral I/R, leading to brain injury (Lakhan et al., 2009; Enzmann et al., 2018). Therefore, there is still a substantial need for the development of therapeutic agents and the elucidation of the pathogenesis and molecular mechanisms of ischemic stroke to improve the functional outcome and prevent recurrence.

Previous research has demonstrated that gene mutation, DNA damage and oxidative stress contribute to the MCAO-induced ischemic stroke (Poudel et al., 2020; Liu et al., 2021; Liu et al., 2022). Recently, an increasing number of studies have indicated that non-coding RNAs (ncRNAs) cause functional alterations in ischemic stroke (Wang et al., 2018; Duan et al., 2019) Non-coding RNAs mainly including miRNAs, long non-coding RNAs (lncRNAs), and circular RNAs (circRNAs) participate in transcriptional regulation at different levels and play vital roles in various physiological and pathological processes(Panni et al., 2020). MiRNAs are small RNA molecules composed of 18–24 nucleotides, functioning in regulation of the expression of the target messenger RNAs (mRNAs), they bind to a short complementary sequence located at the 3’ UTR region of the mRNA and lead to the target mRNA degradation(Ambros, 2004; Lu and Rothenberg, 2018). In an MCAO mice model, it is found that electroacupuncture (EA, a kind of stroke therapy) can reduce neuroinflammation and act in a neuroprotective manner by blocking the miR-223/NLRP3 pathway (Sha et al., 2019). In addition, lncRNAs are defined as transcripts of more than 200 nucleotides which cannot be translated to proteins, they play crucial roles in gene transcription(Ulitsky and Bartel, 2013; Kopp and Mendell, 2018). LncRNA MEG3 promotes cerebral I/R injury through increasing pyroptosis by targeting miR-485/AIM2 axis(Liang et al., 2020) (Chen et al., 2018). The RNA-RNA interplay emerges as a rising star in medical research now, the competing endogenous RNA (ceRNA) are the endogenous RNA transcripts that share the mutual miRNA response elements by competing for the same miRNA pools(Xiao et al., 2020). The ceRNA links different RNA species including mRNA, miRNA, and lncRNA together and enriching our understanding of molecular mechanism of various disease including ischemic stroke.

Thus, we hypothesized that the endogenous RNA regulatory network may be crucial in the emergence of MCAO-induced ischemic stroke. However, there is a lack of integrative analysis of mRNA-miRNA-lncRNA regulatory network in MCAO-induced Ischemic Stroke. To exploit the regulatory mechanism of ischemic stroke formation in this study, we conducted a high-throughput mRNA, miRNA, and lncRNA sequencing among the MCAO group at 3 h, 6h, and 12 h after surgery and the control group. We next explored differentially expressed lncRNA, miRNA and mRNA between these MCAO and control groups. Afterward, the mRNA-miRNA-lncRNA networks were developed to further understand the pathophysiology and underlying molecular mechanisms of MCAO-induced ischemic stroke.

## 2 Materials and methods

### 2.1 Animal experiments

A barrier system houses 10- to 12-week-old C57BL/6J mice with free access to food and water. Zunyi Medical University’s Animal Experimentation Ethics Committee approved all procedures. Based on our previous research (Yao et al., 2016), we occluded the MCA in mice to perform transient MCAO. Anesthesia was induced in mice with sodium pentobarbital (40 mg/kg). There was exposure of the left common artery and the left external carotid artery. Through the right internal carotid artery, a 3 cm long MCAO suture (0.23/0.02 mm head/0.0104 mm body, RWD Life Science, MSMC23B104PK50) was inserted into the middle cerebral artery. Reperfusion was performed after 90 min of occlusion and removal of MCAO sutures. Similar operations were performed on sham control animals to expose the carotid arteries without occluding the middle cerebral artery.

### 2.2 Library preparation for ceRNA sequencing

Each sample contained 1.5 g RNA for removal of rRNA using the Ribo-Zero rRNA Removal Kit (Epicentre, Madison, WI, USA). In order to attribute sequences to each sample, NEBNextR UltraTM Directional RNA Library Prep Kit for IlluminaR (NEB, USA) was used to prepare sequencing libraries. NEBNext First Strand Synthesis Reaction Buffer (5X) was used to carry out fragmentation using divalent cations under elevated temperature. With the assistance of random hexamer primers and reverse transcriptase, first strand cDNA was synthesized (Mao et al., 2021a). DNA Polymerase I and RNase H were then used to synthesize second-strand cDNA. Exonuclease/polymerase activity was used to clear remaining overhangs. For hybridization, NEBNext Adaptor with hairpin loop structure was ligated after adenylation of 3′ ends of DNA fragments. By using AMPure XP beads (Beckman Coulter, Beverly, USA), library fragments were screened for preferred insert fragments that were 150–200 base pairs in length (Cao et al., 2020). Following that, 3 mL of USER Enzyme (NEB, USA) was used with cDNA that was adaptor ligated and size-selected before PCR. After that, PCR was performed with Phusion High-Fidelity DNA polymerase, Universal PCR primers, and Index(X) primers. Finally, the Agilent Bioanalyzer 2,100 and qPCR were used to assess library quality and purify PCR products (AMPure XP system).

### 2.3 Quality control

In-house Perl scripts were used to process raw reads in fastq format. We obtained clean data (clean reads) by removing adapter, ploy-N, and low quality reads from raw data. Sequences longer than 35 nt or smaller than 15 nt were removed from reads before trimming and cleaning. Also, Q20, Q30, and GC-content of the clean data were calculated. By using Cutadapt software (v1.9.1), low-quality reads for base quality under 20 were eliminated. All the downstream analyses were based on clean data with high quality.

### 2.4 mRNA identification

Ensembl database was used to obtain gene annotations and reference genome files. HISAT2 software (v2.0.1) aligned the clean data with the reference genome (Kim et al., 2015). Expression analysis was performed by annotating and indexing the transcripts. RSEM software (v1.2.15) was used to estimate the expression of related genes using an annotated file as a reference gene set.

### 2.5 lncRNA identification

StringTie was used to assemble the transcriptome using reads mapped to the reference genome (Pertea et al., 2015). We annotated the assembled transcripts using the gffcompare program. We differentiated known lncRNAs from assembled transcripts if the sequencing species has lncRNA annotations. Putative lncRNAs were sought for using the remaining unknown transcripts. CPC/CNCI/Pfam/CPAT were combined to sort non-protein codingRNA candidates from putative protein-codingRNAs in the unknown transcripts. Aminimum lengths and exon number thresholds were used to filter out potential protein-coding RNAs. lncRNA candidates with a length of at least 200 nt and more than two exons were selected and further screened using the CPC/CNCI/Pfam/CPAT that differentiate protein-coding and non-coding genes. Additionally, lncRNAs were selected based on their types, such as lincRNA, intronic lncRNA, anti-sense lncRNA, and sense lncRNA.

### 2.6 miRNA identification

Utilizing Bowtie tools software, the Clean Reads database and Rfam database for sequence alignments, filtering out ribosomal RNAs (rRNAs), transfer RNAs (tRNAs), small nuclear RNAs (snRNAs), small nucleolar RNAs (snoRNAs) and repeats, and converting these to ncRNAs. By comparing the reads with known miRNAs from miRBase, we detected known miRNA and novel miRNA predictions(Ni et al., 2022). A novel miRNA secondary structure prediction was performed using Randfold tools software.

### 2.7 DE-ceRNA analysis

We used the R package ‘DESeq2′ to analyze differential expression of ceRNA as well as mRNA, miRNA, and lncRNA(Zhu X. et al., 2022). The ceRNA with *p*-value < 0.05 and log_2_|fold change| ≥ 2 were identified as DE-ceRNAs. Volcano plots and heatmaps were constructed using the pheatmap package and ggplot2 R package(Mao et al., 2021b).

### 2.8 Functional analysis of DE-mRNA

DE-mRNA functions were revealed through Gene Ontology (GO) analysis, including biological processes (BP), cellular components (CC), and molecular functions (MF), along with KEGG pathway enrichment analysis. The R package ‘clusterProfiler’ was used to analyze GO enrichment and KEGG pathway enrichment analyses (Zhang et al., 2020). Those GO categories with adjust. *p*-value<0.05 and KEGG pathways with *p*-value<0.05 were considered as significantly enriched (Mao et al., 2020).

### 2.9 Protein‒Protein interaction (PPI) analysis

DE-mRNA PPI network analysis was carried out based on STRING v11.5 database (https://string-db.org/) (Cao et al., 2021b). Direct interactions between DE-mRNAs were selected. In the PPI network, proteins associated with similar biological processes were grouped according to their attributes (event, betweenness, and degree) (Cao et al., 2021a).

### 2.10 Construction of lncRNA–miRNA‒mRNA regulatory networks

TargetScan (http://www.targetscan.org/vert_72/) and miRanda algorithm were used to establish the lncRNA-miRNA-mRNA networks.

## 3 Results

### 3.1 DE-mRNA screening

**FIGURE 1:**
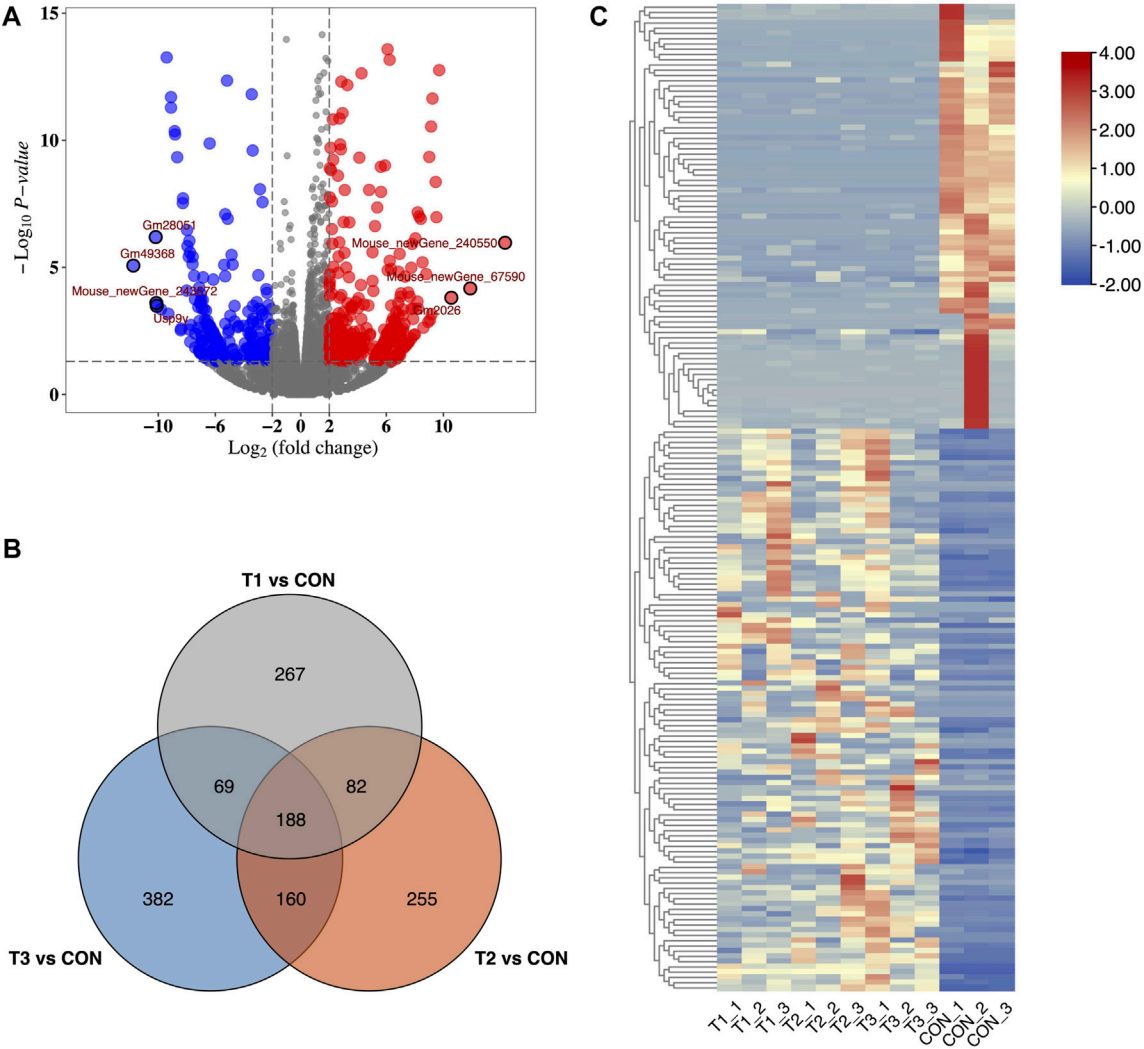
Identification of DE-mRNA in MCAO. **(A)** Volcano plots of DE-mRNAs for 3 h after MCAO treatments. Red and blue dots showed up- and down-regulated DE-mRNAs. **(B)** Heatmap of DE-mRNAs in MCAO and control groups. **(C)** Venn plot of potential DE-mRNA candidates with T1 vs. CON, T2 vs. CON, and T3 vs. CON. T1: 3 h after MCAO, T2: 6 h after MCAO, T3:12 h after MCAO, and CON: control.

We identified 31,011 mRNAs via high-throughput sequencing in at least one treatment (at 3h, 6h, and 12 h after MCAO surgery and control) (Supplementary Table S1). A total of 606, 685, and 799 DE-mRNAs were deemed to be significantly differentially expressed in 3 h vs. control, 6 h vs. control, and 12 h vs. control comparisons, respectively. Differences in mRNA expression in each treatment were evaluated by volcano plot analysis (Figure 1A). Among them, 188 common DE-mRNAs were overlapped in all three treatments which included 86 known mRNAs and 102 novel mRNAs detected in this study (Figure 1B). The common DE-mRNA expression patterns were visualized in a heatmap using hierarchical clustering analysis. (Figure 1C).

### 3.2 Functional analysis of DE-mRNA

**FIGURE 2:**
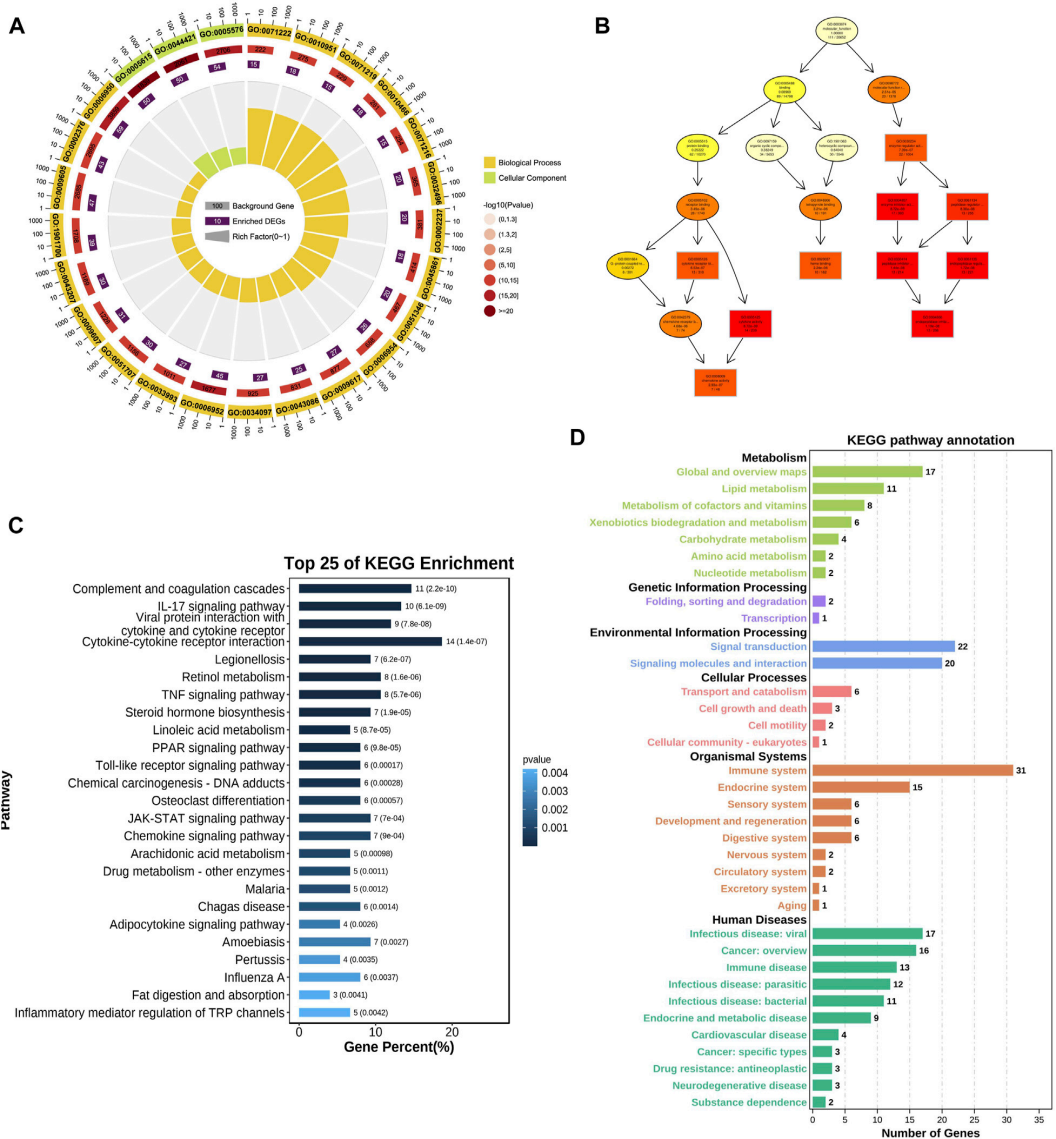
GO and KEGG enrichment analysis of DE-mRNA. **(A)** Top 25 BP GO enrichment analysis results of DE-mRNA. **(B)** GOplot of enriched GO terms **(C)** Top 25 KEGG pathway analysis results of DE-mRNA. **(D)** KEGG pathway annotation results of DE-mRNA. The color of bars represented different KEGG categories.

GO and KEGG analyses were used to reveal the underlying roles of 188 common DE-mRNAs. The top enriched biological process (BP) GO terms included cellular response to lipopolysaccharide (GO:0071222), inflammatory response (GO:0006954), and response to biotic stimulus (GO:0009607) (Figures 2A, B). The main enriched CCs included extracellular space (GO:0005615) and endomembrane system (GO:0012505) (Supplementary Figure S1). The main enriched MFs included cytokine activity (GO:0005125), chemokine activity (GO:0008009), and endopeptidase inhibitor activity (GO:0004866) (Supplementary Figure S2). The top 25 KEGG pathways of common DE-mRNAs were shown in Figure 2C. The DE-mRNAs were primarily enriched in the following pathways: cytokine‒cytokine receptor interaction, tumour necrosis factor (TNF) signaling pathway, JAK-STAT signaling pathway, and complement and coagulation cascades. The main KEGG annotation of common DE-mRNAs were enriched in metabolism global and overview maps, signal transduction, and immune system (Figure 2D).

### 3.3 PPI network analysis

**FIGURE 3:**
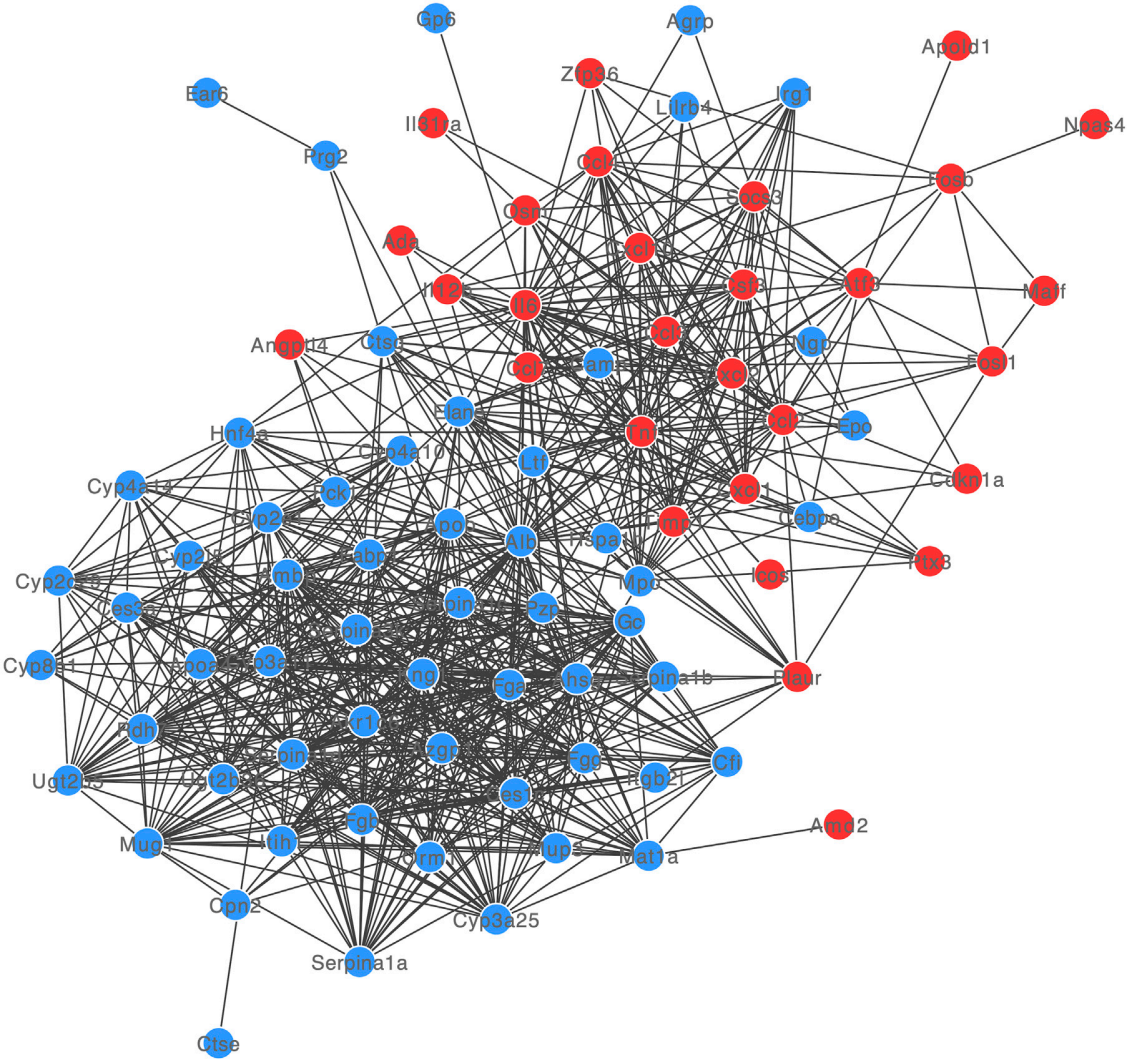
PPI network of DE-mRNAs. Red and blue dots showed up- and down-regulated DE-mRNAs.

The PPI network of DE-mRNA target proteins was established. As shown in Figure 3, this interaction network revealed that the 12 DE-mRNA target proteins showed more than 30° with other proteins. Among them, Albumin had the highest node degree (n = 55) in the network followed by IL-6 and TNF (with node degree of 42 and 41, separately).

### 3.4 Identification of DE-lncRNA and DE-miRNA

**FIGURE 4:**
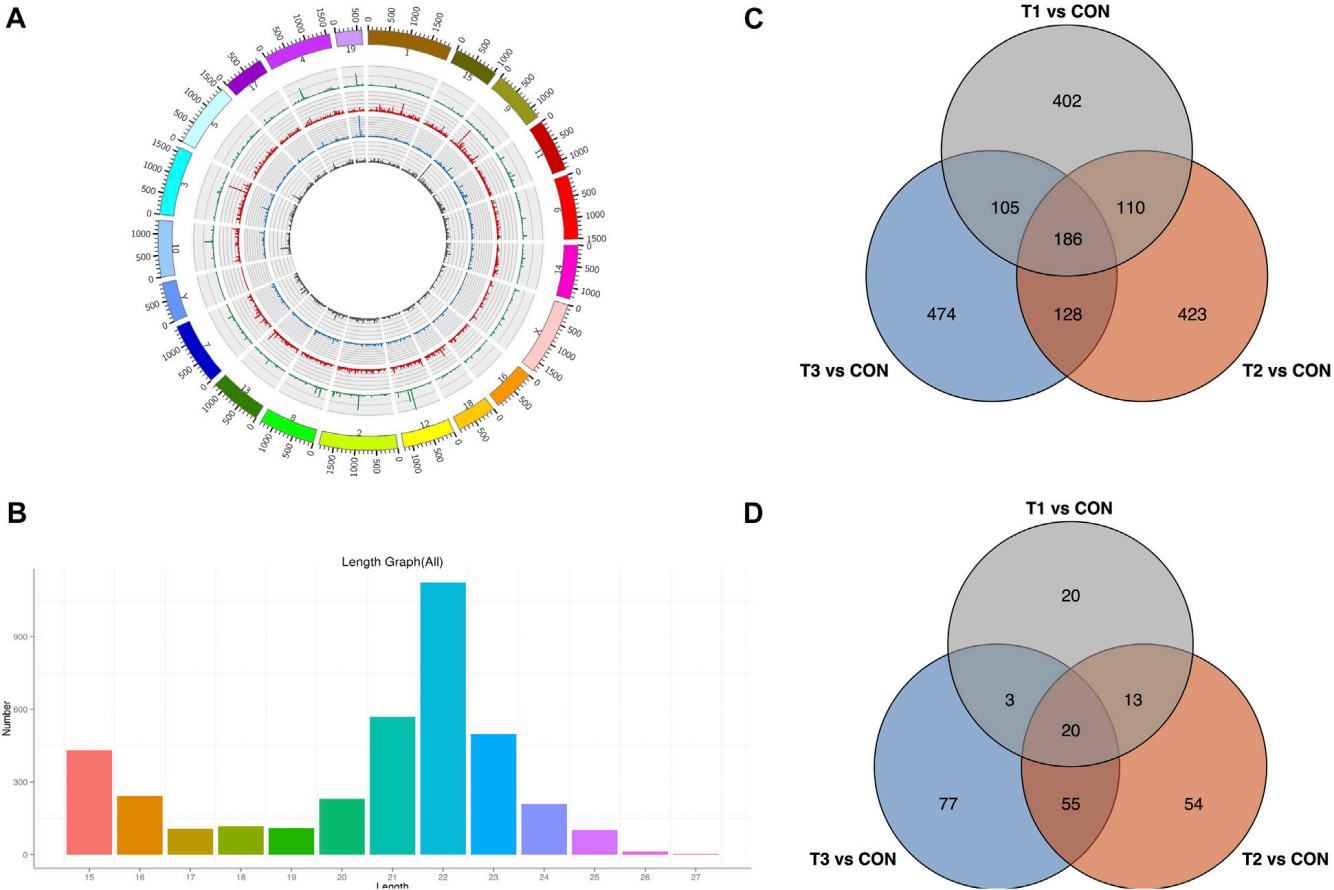
Identification of DE-lncRNA and DE-miRNA. **(A)** Chromosomal distribution of sequenced lncRNA. **(B)** Venn plot of potential DE-lncRNA candidates with T1 vs. CON, T2 vs. CON, and T3 vs. CON. **(C)** Length distribution of sequenced miRNA. **(D)** Venn plot of potential DE-miRNA candidates with T1 vs. CON, T2 vs. CON, and T3 vs. CON. T1: 3 h after MCAO, T2: 6 h after MCAO, T3:12 h after MCAO, and CON: control.

To explore the potential MCAO-related ncRNAs, we sequenced and detected lncRNA and miRNA expression profiles of MCAO using high-throughput sequencing (Figures 4A, B). We detected 4,509 lncRNAs in at least one treatment (at 3h, 6h, and 12 h after MCAO surgery and control) (Supplementary Table S2). A total of 803, 847, and 893 DE-lncRNAs were identified to be significantly differentially expressed with |log2(fold change) |≥2 and *p* < 0.05 in 3 h vs. control, 6 h vs. control, and 12 h vs. control comparisons, respectively. Among them, 186 common DE-lncRNAs were overlapped in all three treatments which included 26 known and 160 novel lncRNA (Figure 4C). Meanwhile, 3,751 miRNAs were identified in at least one treatment (at 3h, 6h, and 12 h after MCAO surgery and control) (Supplementary Table S3). A total of 56, 142, and 155 DE-miRNAs were identified to be prominently differentially expressed in 3 h vs. control, 6 h vs. control, and 12 h vs. control comparisons, respectively. Twenty common DE-miRNA were overlapped in all three treatments which included 2 known miRNAs (mmu-miR-466m-3p and mmu-let-7j) and 18 novel miRNAs detected in this study (Figure 4D).

### 3.5 Construction of the lncRNA–miRNA‒mRNA regulatory network

**FIGURE 5:**
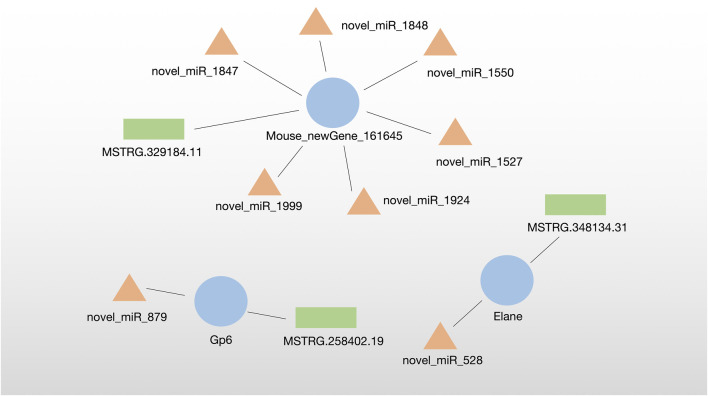
Identified three DE-ceRNA (lncRNA–miRNA‒mRNA) regulatory networks.

The lncRNA–miRNA‒mRNA regulatory network was analyzed by miRanda algorithm and visualized in the network. We identified three DE-ceRNA networks consisting of DE-lncRNA–DE-miRNA‒DE-mRNA. Among them, one network included one new Gene (Mouese_newGene_161645) and six novel miRNAs identified in this sutdy. Moreover, two networks contained known DE-ceRNA as Gp6 (mRNA)-novel_miR_879 (miRNA)-MSTRG.258402.19 (lncRNA) and Elane (mRNA)-novel_miR_528 (miRNA)-MSTRG.348134.31 (lncRNA) (Figure 5).

## 4 Discussion

An ischemic stroke occurs when a blood vessel in the neck or brain is blocked, which is currently a serious threat to human health and life. It is mainly characterized by the blockade of blood flow by an occlusive thrombus. The pathogenesis and molecular mechanism of MCAO remain elusive. Past studies have focused on the key mRNAs and ncRNAs which are essential in the development and curing of MCAO(Hou and Cheng, 2018; Duan et al., 2019). Hereby, we parsed out the mechanisms from another perspective, which focuses on the interactions among different RNAs.

We found DE-mRNAs and built their PPI network, among which Alb, IL-6, and Tnf have the highest node degree. Alb is the abbreviation of albumin, which is the main content of human blood. Several rodent models of ischemic stroke have demonstrated remarkable efficacy with human serum albumin (Huang and Xiao, 2021), it is a potential predictor of pneumonia after an acute ischemic stroke(Bath, 2013). Inflammation is a hallmark of stroke pathology. The interleukin-6 (Il6 or IL-6) is one of the interleukins and a major cytokine, produced by microglia under stroke (Zhu H. et al., 2022). Ischemic stroke pathogenesis is heavily influenced by IL-6 and has been demonstrated as the early marker of acute ischemic stroke(Li et al., 2022; Papadopoulos et al., 2022). Tumour necrosis factor (TNF or Tnf) is best known as a proinflammatory cytokine. After MCAO occurred, microglia produce IL-6 and TNF-α and the microglia-derived TNF-α mediate endothelial necroptosis aggravating blood brain-barrier disruption (Chen et al., 2019).

The top enriched BP GO analysis includes cellular response to lipopolysaccharide (GO:0071222) and inflammatory response (GO:0006954). It has been verified that bacterial lipopolysaccharide is associated with stroke (Hakoupian et al., 2021). Poststroke cognitive impairment is common among stroke patients, and gut microbiota can contribute to it by influencing lipopolysaccharide levels (Wang et al., 2022). In recent years, the post-stroke immune response has emerged as a new breakthrough target in the treatment strategy for ischemic stroke pathobiology and outcome(Xu et al., 2020).

In this study, two ceRNA networks were identified, which contained known DE-ceRNA as Gp6(mRNA)-novel_miR_879(miRNA)-MSTRG.258402.19 (lncRNA) and Elane(mRNA)-novel_miR_528(miRNA)-MSTRG.348134.31 (lncRNA). Gp6, the membrane glycoprotein 6, is a platelet-specific collagen receptor exclusively expressed in the megakaryocytic lineage. Gp6 stimulates platelet activation and adhesion by interacting with collagen which is essential for thrombus formation, causing ischemic stroke (Jung and Moroi, 2008; Gao et al., 2021). A recent study has demonstrated that Gp6 contributes to atherosclerotic cerebral ischemic stroke development by activating the FYN-PKA-pPTK2/FAK1 signaling pathway, indicating the critical roles of Gp6 in ischemic stroke (Gu et al., 2021). Gp6 deficiency or inhibition suppresses thrombus formation and may still not cause a significant bleeding tendency, antibodies such as Abciximab, Glenzobimab and small molecule inhibitors as anti-thrombotic agents have been used for curing stroke clinically (Akkerhuis et al., 2001; Matsumoto et al., 2006; Wichaiyo et al., 2022). Therefore, Gp6 has been verified as a potential target for MCAO-induced ischemic stroke.

In addition, Elane is a neutrophil-expressed gene encoding Elastase. It is well known that neutrophils fight infection by phagocytosis and degranulation. Neutrophils release catalytically active Elane to kill cancer cells instead of non-cancer cells (Cui et al., 2021). Neutrophil extracellular traps (NETs) have been demonstrated to promote thrombus formation (Martinod and Wagner, 2014) and the elevated plasma NET biomarkers correlated with worse stroke outcomes (Denorme et al., 2022). Nets also impair revascularization and vascular remodeling after stroke (Kang et al., 2020). In acute ischemic stroke mice, the Elane inhibitor agaphelin reduces thrombosis, inflammation, and damage to the blood-brain barrier, indicating that Elane is a promising target for ischemic stroke (Leinweber et al., 2021).

In summary, we identified DE-mRNA, DE-miRNAs, and DE-lncRNAs in MCAO using high-throughput sequencing and then established an interplaying regulatory network. We found they work together to regulate MCAO-induced ischemic stroke development. Moreover, our study provided valuable and high-quality ceRNA sequencing data for ischemic stroke research as a reference. On the other hand, there were also several limitations. Firstly, the sample size was limited, which may affect the statistical power of subsequent analyses. Thus, we will further obtain a large number of sample data to validate our results. Secondly, the biological roles of the identified ceRNAs especially novel ceRNAs were not comprehensively explored. In the future, we will verify the regulatory functions through more bioinformatics analysis and experiments to facilitate elucidation of the underlying molecular mechanisms in ischemic stroke occurrence and development.

## Data Availability

The original contributions presented in the study are publicly available. This data can be found here: https://www.ncbi.nlm.nih.gov/sra. Accession number: PRJNA957982.
