# Supplementary figures and images for: Identification of the lncRNA–miRNA‒mRNA regulatory network for middle cerebral artery occlusion-induced ischemic stroke
Source: Front Genet. 2023 May 9;14:1169190. doi: 10.3389/fgene.2023.1169190 (PMC10203218; doi:10.3389/fgene.2023.1169190)

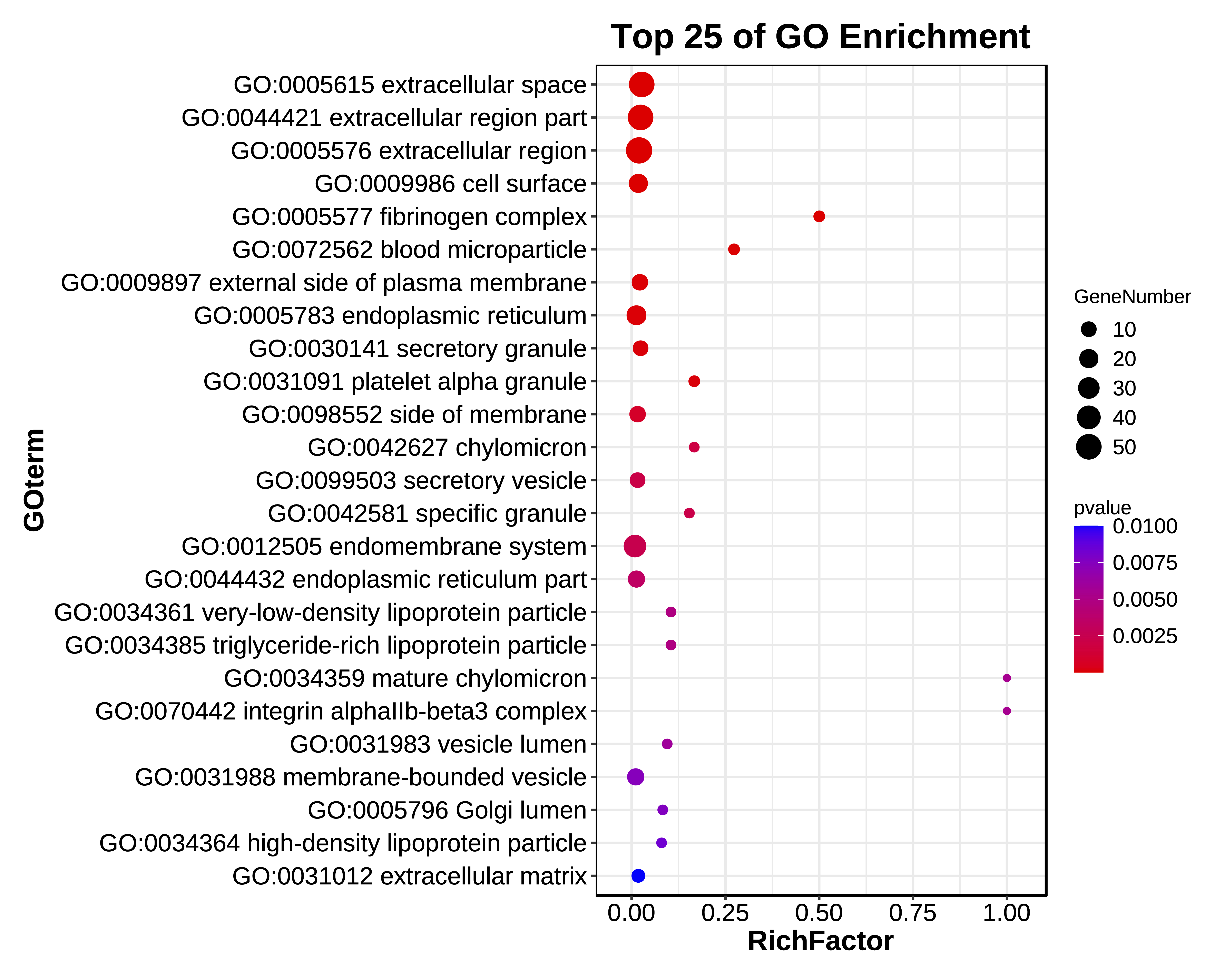

Supplement: Supplementary file 3 [file Image1.JPEG]

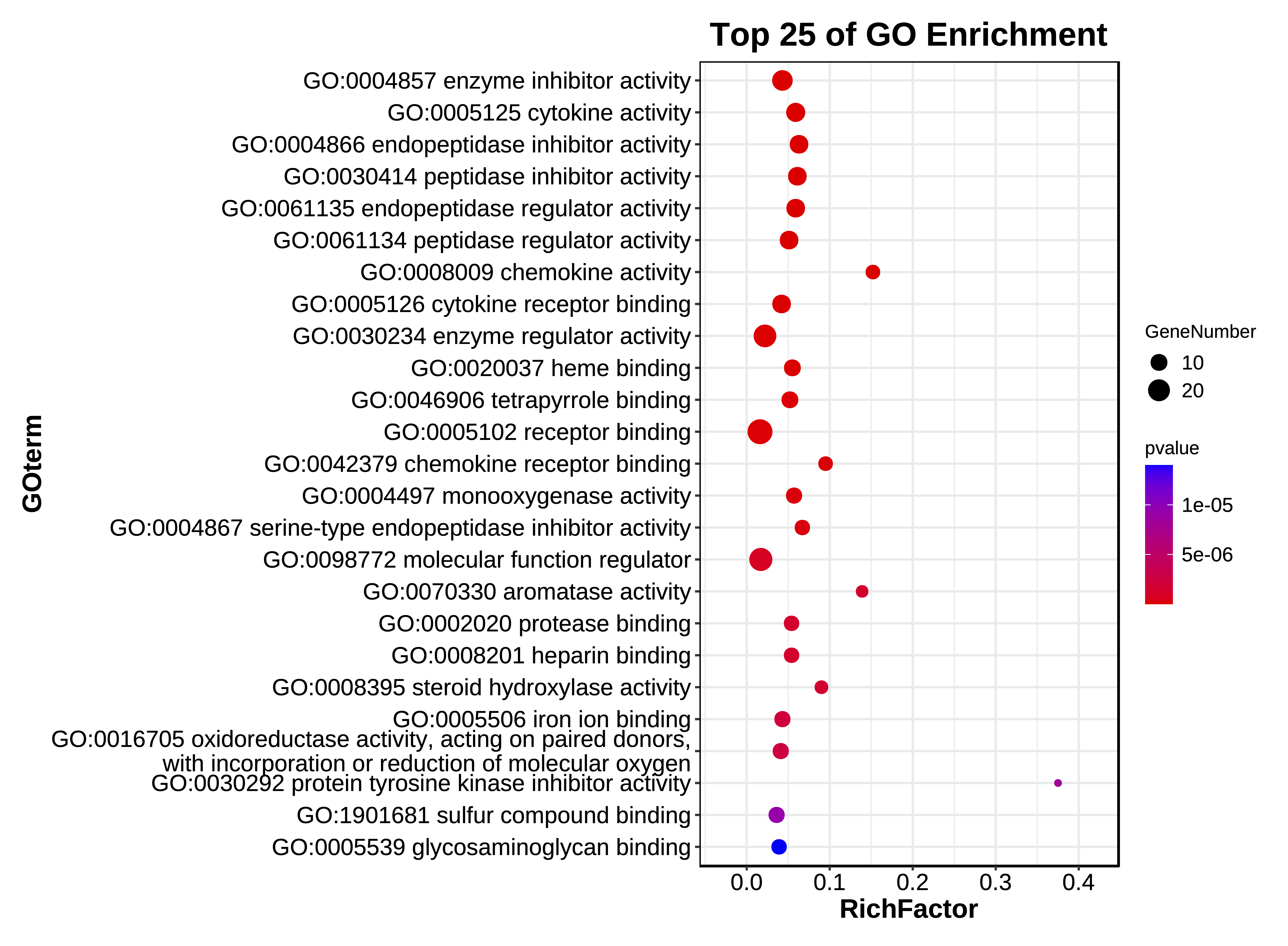

Supplement: Supplementary file 5 [file Image2.JPEG]
